# Use of rituximab and plasma exchange in treatment and prophylaxis of recurrent FSGS

**DOI:** 10.1093/ckj/sfaf058

**Published:** 2025-02-20

**Authors:** Sophie Gharaei, Julia Gharaei, Omar Ragy, Durga A K Kanigicherla

**Affiliations:** Manchester Medical School, University of Manchester, Manchester, UK; Manchester Institute of Nephrology and Transplantation, Manchester, UK; School of Biomedical and Health Sciences, Universidad Europea de Madrid, Madrid, Spain; Manchester Institute of Nephrology and Transplantation, Manchester, UK; University of Manchester, Manchester Academic Health Science Centre, Manchester, UK; Manchester Institute of Nephrology and Transplantation, Manchester, UK; University of Manchester, Manchester Academic Health Science Centre, Manchester, UK

**Keywords:** focal segmental glomerulosclerosis, plasma exchange, rituximab, transplantation

## Abstract

**Background:**

Focal segmental glomerulosclerosis (FSGS) is a common cause of nephrotic syndrome and renal failure, requiring transplantation. However, FSGS can often recur after transplantation resulting in graft failure. The most used therapeutic intervention for rFSGS is plasma exchange (PE), with variable success. Recently, rituximab has found increasing use in both treatment and prevention of recurrent FSGS.

**Methods:**

We undertook a systematic review of therapeutic ± preventative plasma exchange, rituximab or a combination of both for recurrent FSGS. Studies published between 2017 and 2024 were included, to reflect the most contemporary clinical practice.

**Results:**

Twenty-seven studies with a total of 475 patients received treatment for recurrence post-transplantation and/or for prevention of recurrent FSGS. Of 221 patients who received plasma exchange on its own as therapy, 156 (71%) achieved either complete or partial remission. Rituximab alone was used in only four patients (75% remission rate), while 67% achieved remission with a combination of both. One hundred and forty-two patients received pre/peri-transplantation treatment to prevent recurrence in the graft. Fifty-one patients (36%) experienced recurrence despite prophylaxis. Recurrence rates were 35% with plasma exchange alone and 38% with rituximab alone*.*

**Conclusion:**

We conclude that rituximab did not add significant benefit to plasma exchange when used as initial therapeutic intervention in post-transplant recurrent FSGS. The modest benefit of prophylactic therapies highlights the need for risk stratification to identify patients most likely to benefit from such interventions. Larger prospective studies with standardized approaches to treatment are essential in improving outcomes in rFSGS.

KEY LEARNING POINTS **What was known:**

- Focal segmental glomerulosclerosis (FSGS) often recurs after transplantation despite prophylactic intervention and is difficult to treat.
- Plasma exchange is used most commonly, with variable success rates.
- Rituximab has been used for treatment and prevention, either alone or in combination with plasma exchange; however, evidence on effectiveness remains conflicting.

**This study adds:**

- Our study shows that plasma exchange alone or in combination with rituximab results in similar remission rates. Use of rituximab in isolation was reported in only four patients.
- Rituximab showed similar benefit to prophylactic plasma exchange in prevention of recurrent FSGS; however, reports of this approach are limited.
- There are very sparse reports to appreciate the effect of preventative measures on treatment responsiveness following subsequent recurrence.

**Potential impact:**

- Our study is the first systematic review that directly compares outcomes between the different intervention regimes using plasma exchange and rituximab in treatment, prophylaxis, and treatment responsiveness after prior prophylaxis. We found no significant difference in remission rates and suggest that clinical practice should be guided by individual patient risk, tolerability, local availability, and cost until robust evidence is available.
- Combination of plasma exchange and rituximab showed no clear advantage over plasma exchange alone and its use could be limited to specific patient subgroups, such as those with severe or refractory disease.
- Larger prospective controlled studies with specific treatment protocols and longer follow up are desperately needed to evaluate the best approach in treatment and prevention of recurrent FSGS.

## INTRODUCTION

Focal segmental glomerulosclerosis (FSGS) is a common cause of nephrotic syndrome in both adults and children, often progressing to renal failure, and can recur after kidney transplantation (recurrent FSGS, [rFSGS]). Despite treatment, rFSGS frequently can lead to graft failure. It is believed that circulating factors affecting podocytes and glomerular permeability may contribute to the pathogenesis of primary FSGS. This constitutes the basis for the use of plasma exchange (PE), as it is believed to remove the pathogenic factors, thus inducing remission [1]. In native kidney FSGS first-line treatment remains steroid therapy, with up to 63% of patients achieving remission [2]. Aside from the adverse effects of long-term steroid use, it has been reported that over 50% of patients experience at least one relapse within 12 months [3], highlighting the importance of investigating alternative therapies.

In recent years, rituximab (RTX), a monoclonal, chimeric antibody that binds CD20+ B-lymphocytes, has found increasing use in the prevention and treatment of recurrent FSGS. It is hoped that the combination of PE and RTX may not only induce remission but may also offer a viable long-term option in steroid-resistant FSGS [4]. Furthermore, it has been hypothesized that RTX may reduce the need for other immunosuppressive therapies, as well as allowing a more rapid discontinuation of PE therapy, which is associated with its own adverse effects [5].

This systematic review was conducted to evaluate the latest evidence on efficacy of RTX and PE in the prevention and treatment of recurrent FSGS post-transplantation.

## MATERIALS AND METHODS

A literature search was conducted to identify studies from 2017 to 2024 that included adult and paediatric patients with a diagnosis of recurrence of primary FSGS in their renal transplant, who received treatment with PE, RTX, or a combination of both, and/or have received prophylactic treatment with PE or RTX ± PE before transplantation.

**Figure 1:**
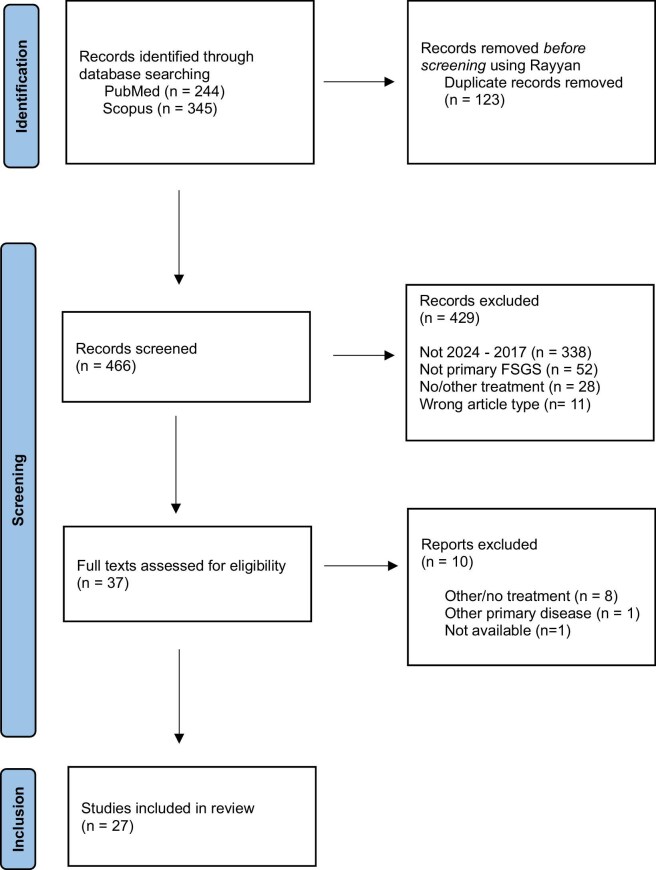
PRISMA 2020 flow diagram of search methodology.

The first searches in both PubMed and Scopus databases included the search terms ‘FSGS’, ‘focal segmental glomerulosclerosis’, ‘nephrotic syndrome’, ‘podocytopathy’, ‘recurrence’, ‘relapse’, ‘transplant’, ‘allograft’ ‘rituximab’, ‘monoclonal antibody’, and ‘plasmapheresis’. After applying the filters full text, English language, and articles, 244 and 345 results were found, respectively. The articles were selected as outlined in Fig. 1.

Twenty-seven studies with a total of 475 patients were included.

### Review process

Two reviewers conducted the eligibility screening independently, excluding studies with (i) patients whose diagnosis in the native kidneys was not primary FSGS, (ii) patients who received no or other treatments than PE and RTX, and (ii) studies that were not primary research. Two reviewers then discussed any conflicts and mutually agreed on inclusion or exclusion of the papers.

### Meta-analysis

**Figure 2:**
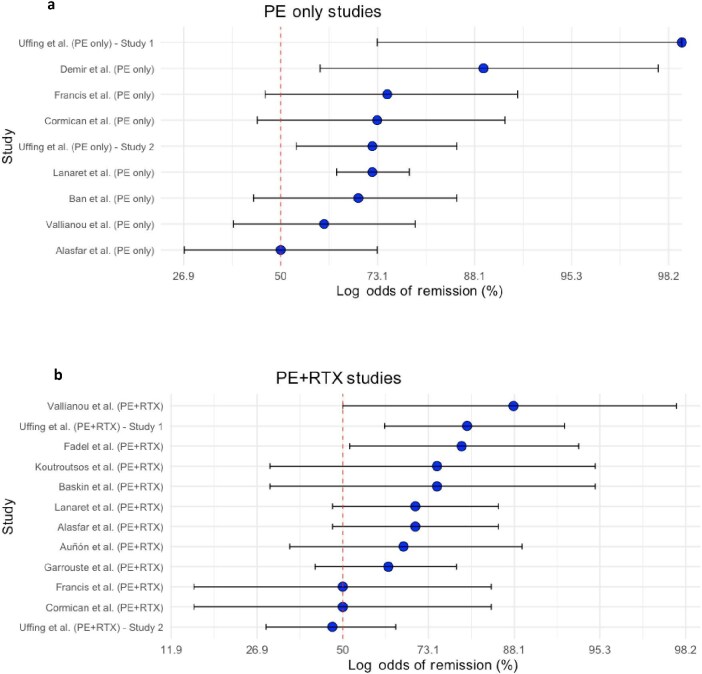
(**a**) Forest plots illustrating odds of remission (%) for pooled studies using PE only as treatment. (**b**) Forest plots illustrating odds of remission (%) for pooled studies using PE + RTX as treatment.

We evaluated the effectiveness of various therapeutic and prophylactic strategies across multiple studies in meta-analysis (Fig. 2a, b and Table 5a, b). Studies were analysed by calculating the recurrence or remission rates, with proportions expressed as a measure of effect size. Confidence intervals (CIs) for these proportions were calculated using the Wilson score method to ensure robust estimates. Therapeutic studies were pooled into two categories: PE (PE only) and PE combined with RTX (PE + RTX). These categories were analysed and displayed in separate forest plots to illustrate remission outcomes. Studies using prophylactic treatment were similarly assessed, with their recurrence rates calculated and displayed in an additional plot. In plots depicting recurrence after prophylaxis, values >50% represent a higher likelihood of negative outcomes, whereas in plots showing remission after treatment values >50% indicate improved therapeutic outcomes. Recurrence or remission rates (proportions) were calculated directly, and logit transformations were applied to normalize the distributions, as proportions often deviate from normality. The confidence intervals were also logit-transformed and displayed on forest plots, enabling visualization of study-specific proportions and their variability. These analyses were conducted using R version 1.4.1717.

## RESULTS

A total of 466 papers underwent title and abstract screening, 429 of which were excluded based on the criteria outlined above. Thirty-seven papers underwent full-length review, resulting in 10 exclusions and a total of 27 papers being included for data extraction. Data were extracted from all patients that experienced recurrence after transplantation and had received either pre-transplant treatment as prevention, therapy for the recurrence, or both. Recurrence of FSGS was determined on biopsy or clinically with recurrence of proteinuria, often nephrotic range, definitions ranging from >0.5 to >3 g/day. In most studies, complete remission (CR) was defined as 24-hour proteinuria <0.5 g. Partial remission (PR) was defined as proteinuria that remained in sub-nephrotic range or a reduction by 50% compared with known peak values. No remission (NR) was reported when there was no or less than half the reduction of peak proteinuria.

### Treatment of post-transplant FSGS

Treatment outcomes are summarized in Table 1 for the total cohort. A total of 475 patients across the 27 studies received pre- and/or post-transplantation treatment for recurrent FSGS. The studies used are summarized in Tables 2 and 4. The majority of patients (69%) achieved some form of remission on therapy after rFSGS. Graft loss within 10 years due to recurrence after transplantation was reported for 58 patients in total, most of whom had not or only partially responded to treatment. Improvement in proteinuria (complete or partial response) was reported in 71% of patients on PE only, and 67% of patients who received PE and RTX together. The number of patients receiving RTX only treatment was significantly smaller (*n* = 4), with a remission rate of 75%. One hundred and twenty-two of 397 patients (31%) achieved NR.

**Table 1:** Cumulative summary of treatment outcomes from included studies.

Treatment	Complete remission	Partial remission	No remission	Total
PE only	73 (33)	83 (38)	65 (29)	221
RTX only	2 (50)	1 (25)	1 (25)	4
PE + RTX	50 (29)	66 (38)	56 (33)	172
Total	125 (31)	150 (38)	122 (31)	397

Values are total numbers of patients and proportions (%).

**Table 2:** Summary of studies reporting outcomes after treatment alone.

Authors	Methods	Patients with recurrence	Age at primary diagnosis	Time to ESKD	Age at transplant	Intervention	Treatment outcome
Kienzl-Wagner *et al*. [6]	Case report	1 male	NA	NA	5 years	PE + RTX, *n* = 1	NR Graft loss
Solomon *et al*. [7]	Case report	1 male	8 years	3 years	11 years	PE + RTX, *n* = 1	PR
Al-Jehani *et al*. [8]	Case report	1 female	10 years	NA	13 years	PE + RTX, *n* = 1	CR
Halfon *et al*. [9]	Case report	1 patient	NA	NA	43 years	PE + RTX, *n* = 1	NR
Argiolas *et al*. [10]	Case report	1 patient	30 years	4 years	54 years	PE + RTX, *n* = 1	CR
Kim *et al*. [11]	Case report	1 patient	35 years	NA	55 years	PE + RTX, *n* = 1	PR
Colucci *et al*. [12]	Case reports	2 males	18 months/7 years	8.5/5 years	Both at 15 years	PE + RTX, *n* = 2	1 PR, 1 NR
Ramirez-Guerrero *et al*. [13]	Case reports	1 male, 2 females	NA	0/9/2 years	53/47/47 years	PE + RTX, *n* = 3	3 PR
Koutroutsos *et al*. [14]	Retrospective cohort study	4 patients	NA	NA	55/41/57/49 years	PE + RTX, *n* = 4	1 CR, 2 PR, 1 NR 1 graft loss
Demir *et al*. [15]	Prospective single-centre case series	9 patients	15–59 years	1–7 years	Youngest 16 years, oldest 61 years	PE only, *n* = 9	2 CR, 6 PR, 1 NR 3 graft losses (1 due to infection)
Cormican *et al*. [16]^a^	Retrospective cohort study (national)	16 patients	Mean 24.4 years	5.5 months	Mean 29.5 years	PE only, *n* = 11 PE + RTX, *n* = 4 RTX only, *n* = 1	3 CR, 5 PR 3 NR 2 CR, 2 PR 1 NR 9 graft losses
Garrouste *et al*. [17]	Multicentre retrospective study	19 patients	Median 24 years	NA	Youngest 15 years, oldest 66 years	PE + RTX, *n* = 19	9 CR, 3 PR, 7 NR 4 graft losses during follow-up of 10 years
Francis *et al*. [18]	Retrospective multicentre cohort study	12 patients	NA	2–6 years	Youngest 2 years, oldest 16 years	PE only, *n* = 8 PE + RTX, *n* = 4	6 CR, 2 NR 2 CR, 2 NR

ESKD, end-stage kidney disease; KT, kidney transplant.

a Three patients in this study received abatacept alone or in combination with PE ± RTX and were excluded.

### Preventative therapy prior to/soon after transplantation

Of the 27 studies analysed, 14 studies used prophylactic intervention prior to transplantation to prevent the recurrence of FSGS in the graft. The cumulative results are summarized in Table 3 and individual studies in Table 4. Three of these studies included patients who had been selected for their recurrence, hence they were excluded in our analysis of the efficacy of prophylactic treatments. Fifty-one out of 142 patients (36%) experienced recurrence of FSGS despite prophylactic therapy. Of the patients that received PE only prior to transplantation, 35% experienced recurrence compared with 38% that were treated with RTX only. Only 1 patient received prophylactic treatment with combined PE and RTX and this patient developed recurrence.

**Table 3:** Summary of outcomes of prophylactic therapies.

Prophylaxis	Recurrence	No recurrence	Total
PE only	42 (35)	78 (65)	120
RTX only	8 (38)	13 (62)	21
PE + RTX	1 (100)	0 (0)	1
Total	51 (36)	91 (64)	142

Values are total numbers of patients and proportions (%).

Cohort from Alasfar *et al*. [4] was not included because recurrences (*n* = 23) were noted in 37 patients (PE + RTX, *n* = 28 and RTX only, *n* = 9), but it was not possible to identify recurrences after individual treatment categories.

**Table 4:** Summary of studies used that involved prophylaxis and therapy.

				Intervention		
Authors	Methods	Patients	Transplant	Prophylaxis	Treatment	Treatment outcome
Ural *et al*. [19]	Case report	1 male Age at diagnosis: 38 years Time to ESKD: 6 years	Age at KT: 44 years	PE only, *n* = 1 Recurred	PE + RTX, *n* = 1	NR
Mühlbacher *et al*. [20]	Case report	1 male Age at diagnosis: 17 years Time to ESKD: 2 years	Age at KT: 19 years	RTX only, *n* = 1 Recurred	PE + RTX, *n* = 1	NR
Ino *et al*. [21]	Case report	1 female Age at diagnosis: 15 years	Time to ESKD: 13 years Age at KT: 28 years	PE + RTX , *n* = 1 Recurred	PE + RTX, *n* = 1	CR (additional RTX to maintain remission)
Jain *et al*. [22]	Case report	1 female Age at diagnosis: 15 years	Time to ESKD: 1 year Age at KT: 16 years	PE only, *n* = 1 Recurred	PE + RTX, *n* = 1	NR
El Khashab *et al*. [23]	Prospective short-term observational study	8 patients	Time to ESKD: median 40 months Age at KT: 17–36 years	RTX only, *n* = 8 1 recurred	PE + RTX, *n* = 1	PR
Baskin *et al*. [24]	Retrospective cohort study	34 patients Age at diagnosis: mean 12.7 years	Time to ESKD: mean 4 years Age at KT: mean 11.2 years	PE only, *n* = 34 5 recurred	PE only, *n* = 1 PE + RTX, *n* = 4	NR 3 CR, 1 PR
Fadel *et al*. [25]	Retrospective observational cohort study	28 patients	Age at KT: mean 9.21 years	PE only, *n* = 28 10 recurred	PE + RTX, *n* = 10	8 CR, 2 NR 2 graft losses
Auñón *et al*. [26]	Multicentre retrospective cohort study	19 patients Age at diagnosis: median 24.5 years	Time to ESKD: mean 6.17 years Age at KT: mean 36.1 years	RTX only, *n* = 12 6 recurred	PE only, *n* = 3 PE + RTX *n* = 9 RTX only, *n* = 1	2 PR, 1 NR 1 CR, 5 PR, 3 NR 1 CR
Ban *et al*. [27]^a^	Single centre retrospective study	16 patients Age at diagnosis: 2.1–7.0 years	Time to ESKD: median 3.0 years Age at KT: 8.1–16.6 years	PE only, *n* = 9 PE + RTX, *n* = 3 (selected for recurrence)	PE only, *n* = 13 PE + RTX, *n* = 3	2 CR, 7 PR, 4 NR 1 PR, 2 NR 5 graft losses due to recurrence (10 years)
Vallianou *et al*. [28]	Retrospective single centre study	44 patients Age at diagnosis: mean 21.8 years	Time to ESKD: 15.4–110.7 months Age at KT: mean 33.8 years	PE only, *n* = 34 16 recurred	PE only, *n* = 18 PE + RTX, *n* = 8	5 CR, 6 PR, 7 NR 2 CR, 5 PR, 1 NR 15 graft losses
Uffing *et al*. [29]^a^	Multicentre retrospective case series	27 patients	Time to ESKD: 36–96 months Age at KT: 27–49 years	PE only, *n* = 6 RTX only, *n* = 1 (selected for recurrence)	PE only, *n* = 6 PE + RTX, *n* = 21	3 CR, 3 PR 10 CR, 7 PR,4 NR 5 graft losses
Alasfar *et al*. [4]	Observational prospective cohort study	38 patients Age at diagnosis: mean 30 years Time to ESKD: mean 4 years	Age at KT: mean 38 years 24 had a prior transplant	PE + RTX, *n* = 28 RTX only, *n* = 9 23 recurrences* (not included in analysis)	PE only, *n* = 18 PE + RTX, *n* = 20	9 PR, 9 NR 14 PR, 6 NR
Uffing *et al*. [30]	Observational multicentre cohort study	57 patients Age at diagnosis: 17–43 years	Time to ESKD:15–62 months Age at KT: 28–49 years	PE only, *n* = 22 9 recurred	PE only, *n* = 25 PE + RTX, *n* = 30 RTX only, *n* = 2	7 CR, 11 PR, 7 NR 5 CR, 9 PR, 16 NR 1 CR, 1 PR 22 graft losses
Lanaret *et al*. [5]^a^^,^^b^	Retrospective multicentre study	129 patients Age at diagnosis: mean 25.4 years	Age at KT: mean 39.9 years 42 had a prior transplant	RTX only, *n* = 19 (selected for recurrence)	PE only, *n* = 109 PE + RTX, *n* = 20	45 CR, 34 PR, 30 NR 14 remissions^a^, 6 NR

ESKD, end-stage kidney disease; KT, kidney transplant.

a These studies reported on patients with recurrence and meant that subjects were selected (despite prophylaxis); therefore, they were not included in the analysis of prophylactic strategies.

b In this study of investigation of remission after PE + RTX, *n* = 20, remissions were not categorized as partial or complete and therefore these are considered as partial remissions in the summary table (Table 1).

In studies included in this systematic review, the time between recurrence and start of RTX therapy varied from 1 day after transplantation to >3 months after recurrence. In addition, the doses and frequency administered varied between patients and the studies. Standard dose of RTX is considered 375 mg/m^2^ body surface area; however, some patients received 500 mg/m^2^ or more in a single dose. All studies used one to three doses of RTX. No significant differences in outcomes were observed.

### Meta-analysis

The majority of patients receiving these therapeutic interventions achieved remission, although confidence intervals varied widely between studies. The sample sizes ranged widely, with remission rates displaying considerable variability across studies (Table 5a, b and Fig. 2).

**Table 5a:** Studies involving PE alone for treatment.

Study	Sample size	Remissions	Proportion	95% CI
Alasfar *et al*. [4]	18	9	0.50	0.27–0.73
Lanaret *et al*. [5]	109	79	0.72	0.64–0.79
Demir *et al*. [15]	9	8	0.89	0.60–0.98
Cormican *et al*. [16]	11	8	0.73	0.44–0.91
Francis *et al*. [18]	8	6	0.75	0.46–0.92
Ban *et al*. [27]	13	9	0.69	0.43–0.86
Vallianou *et al*. [28]	18	11	0.61	0.38–0.80
Uffing *et al*. [29]	6	6	1.00	0.73–1.00
Uffing *et al*. [30]	25	18	0.72	0.54–0.86

Studies reporting ≤3 patients in each treatment arm are not included in this meta-analysis.

**Table 5b:** Studies involving combined PE and RTX for treatment.

Study	Sample size	Remissions	Proportion	95% CI
Koutroutsos *et al*. [14]	4	3	0.75	0.30–0.95
Cormican *et al*. [16]	4	2	0.50	0.15–0.85
Garrouste *et al*. [17]	19	12	0.63	0.42–0.79
Francis *et al*. [18]	4	2	0.50	0.15–0.85
Baskin *et al*. [24]	4	3	0.75	0.30–0.95
Fadel *et al*. [25]	10	8	0.80	0.52–0.94
Auñón *et al*. [26]	9	6	0.67	0.35–0.89
Vallianou *et al*. [28]	8	7	0.88	0.50–0.98
Uffing *et al*. [29]	21	17	0.81	0.62–0.93
Alasfar *et al*. [4]	20	14	0.70	0.47–0.86
Uffing *et al*. [30]	30	14	0.47	0.29–0.65
Lanaret *et al*. [5]	20	14	0.70	0.47–0.86

Studies reporting three or fewer patients in each treatment arm are not included in this meta-analysis.

**Figure 3:**
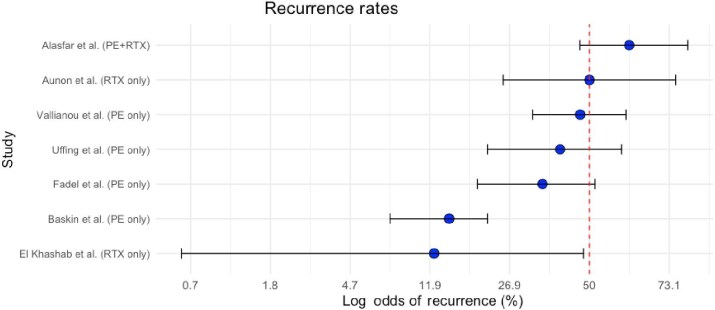
Forest plot illustrating odds of recurrence (%) after prophylactic approaches to prevent rFSGS.

Recurrence rates after prophylactic interventions showed considerable variation across studies, reflecting differences in the strategies and potentially patient factors (Table 6 and Fig. 3). The majority of studies investigating recurrence rates after prophylactic measures reported rates below 60%, with one study reporting as low as 12.5%, highlighting the variability in the effectiveness of these interventions.

**Table 6:** Studies involving prophylactic approaches to prevent recurrent FSGS.

Study	Number of patients	Recurrence	Recurrence rate (%)	95% CI (%)
El Khashab *et al*. (RTX only) [23]	8	1	12.5	0.64%–46.9%
Baskin *et al*. (PE only) [24]	34	5	14.7	6.5%–30.0%
Fadel *et al*. (PE only) [25]	28	10	35.7	20.5%–54.1%
Auñón *et al*. (RTX only) [26]	12	6	50.0	25.4%–74.6%
Vallianou *et al*. (PE only) [28]	34	16	47.1	32.2%–62.3%
Alasfar *et al*. (PE + RTX) [4]	37	23	62.2	46.5%–75.8%
Uffing *et al*. (PE only) [30]	22	9	40.9	23.5%–61.5%

### Therapeutic responsiveness after prophylaxis

Of the 15 studies that used prophylactic therapies, 8 reported outcomes of therapeutic measures after patients developed recurrence. All the 22 patients for whom the data could be extracted received PE, with or without RTX, for their recurrence. Six patients (27%) achieved NR after therapeutic measures, while 73% responded to therapy after recurrence. Most patients (18 out of 22) had received prophylactic PE and a combination of PE and RTX as therapy, which resulted in 11 CRs, 2 PRs and 5 NRs.

## DISCUSSION

As the pathogenesis of FSGS is not yet fully understood, there are no known therapies that reliably target the cause of the disease. The management of rFSGS after kidney transplantation remains a significant challenge for clinicians and patients, given its high recurrence rates and the potential for graft loss. This systematic review analysed 27 studies with data of 475 patients with FSGS who had received therapy for recurrence or prevention of recurrence and provides an overview of treatment outcomes associated with PE and RTX, either alone or on combination.

Our review demonstrates that treatment with PE, RTX, or their combination leads to variable rates of remission, with 69% of patients achieving some form of response. CR was observed in 31% of cases, while 38% attained PR. Importantly, 31% of patients experienced NR, underscoring the heterogeneity in therapeutic responsiveness among individuals with rFSGS. The combination of PE and RTX did not significantly outperform PE alone in achieving remission, with remission rates of 67% and 71%, respectively. However, RTX alone, used in a limited subset of patients (*n* = 4), showed a relatively higher remission rate (75%), though the small sample size precludes definitive conclusions. This suggests that there may not be added benefit to using both interventions together over either on its own as treatment.

PE remains a cornerstone of rFSGS management, particularly in the early post-transplant period. By removing putative circulating permeability factors implicated in podocyte injury, PE has demonstrated efficacy in achieving remission in many patients. However, the need for repeated sessions and associated risks, including infections and vascular access complications, may necessitate adjunctive therapies to enhance its efficacy and reduce its burden. Graft loss due to rFSGS recurrence was reported in 58 patients across the included studies, with the majority of losses occurring in patients who achieved NR. This finding underscores the critical importance of achieving remission in preserving graft function. The results also highlight the need for early identification and aggressive management of recurrence to improve long-term outcomes.

Our review found that 36% of patients experienced recurrence despite prophylactic therapy, with similar recurrence rates for PE (35%) and RTX (38%). This finding suggests that while prophylaxis may reduce recurrence risk, it does not eliminate it, and careful post-transplant monitoring remains essential. The efficacy of combined prophylactic therapy (PE + RTX) could not be evaluated due to the limited number of patients receiving this regimen and the number of patients receiving RTX alone prophylaxis was also relatively low (*n* = 21). The data available are often difficult to interpret due to concomitant prophylactic PE use, the small numbers of patients treated prophylactically with RTX alone, and the lack of identified factors that could predispose patients at higher risk of recurrence post-transplantation. The recent study by Uro-Coste C *et al*. [31] on prophylactic treatment of FSGS recurrence was excluded due to their selection of patients who had relapsed in a previous graft already, thus deeming them as particularly high-risk and not representative of the general patient population in question.

Additionally, it remains unclear whether the decrease of recurrence can be attributed entirely to RTX, due to the lack of a large control group that has only been treated with prophylactic RTX (only 21/142 patients in our review). Furthermore, it has been hypothesized that it is not the RTX on its own that potentially leads to less recurrence when used prophylactically, but rather its potentiating effect on PE, as suggested by Auñón *et al*. [26]. Lanaret *et al*. [5] found that responders to RTX had better native kidney function for longer before transplantation than non-responders. Moreover, they observed that the use of immunosuppressive therapy in the native kidneys reduces the probability of responsiveness to treatment post-transplantation. Furthermore, it has been observed that some patients on RTX are at risk of developing secondary RTX resistance as a result of anti-chimeric antibodies developing in patients exposed to repeated RTX doses [12].

In interpreting these results, the limitations of the review need to be taken into consideration. This review was limited to articles published between 2017 and 2024. The heterogeneity in study designs, patient populations, and treatment protocols limits the generalizability of the findings. Additionally, the reliance on retrospective studies and case reports introduces the potential for selection and reporting biases. Due to variations in local guidelines and policies, treatment practices were not standardized and definitions of recurrence and remission varied slightly. The sample size of many studies was small, notwithstanding the case reports that were part of this review. This is especially the case with the use of therapeutic and prophylactic RTX on its own, as it was used much less in isolation. The timing, dosage, and frequency of RTX administration varied significantly, ranging from single doses to repeated infusions. Similarly, the frequency and duration of PE sessions differed widely. Owing to significant heterogeneity between studies and the fact that there were only four patients who received RTX alone treatment for rFSGS, no formal statistical comparison could be undertaken to compare the different approaches. Despite these limitations, we believe that use of studies published in recent times contemporaneous to current clinical practice provides illustration of treatment and prophylactic responses as well as challenges faced by the nephrology community in this area of practice. Until future research with well-designed prospective studies to provide more robust evidence is available, this systematic review of studies with specific treatments put together gives clinicians and patients tools to make informed choices of individual treatments in rFSGS.

## CONCLUSIONS

In conclusion, this review highlights the potential of PE and RTX in achieving remission in rFSGS after transplantation. Addition of therapeutic RTX to PE has not been found to add significant improvement response after treatment for rFSGS. While prophylactic strategies may be of benefit, their efficacy is limited, and post-transplant monitoring remains critical. There is little literature on treatment responsiveness after prior prophylactic intervention. Larger prospective controlled studies with standardized protocols are essential in providing clearer answers as to the role of these regimes and improving outcomes in recurrent FSGS after transplantation.

## Data Availability

The data supporting the findings of this study are all included within this manuscript and freely available on PubMed and Scopus through respective access.
